# Estrogen Receptor Beta and 2-arachidonoylglycerol Mediate the Suppressive Effects of Estradiol on Frequency of Postsynaptic Currents in Gonadotropin-Releasing Hormone Neurons of Metestrous Mice: An Acute Slice Electrophysiological Study

**DOI:** 10.3389/fncel.2016.00077

**Published:** 2016-03-29

**Authors:** Flóra Bálint, Zsolt Liposits, Imre Farkas

**Affiliations:** ^1^Laboratory of Endocrine Neurobiology, Institute of Experimental Medicine, Hungarian Academy of SciencesBudapest, Hungary; ^2^Roska Tamás Doctoral School of Sciences and Technology, Faculty of Information Technology and Bionics, Pázmány Péter Catholic UniversityBudapest, Hungary; ^3^Department of Neuroscience, Faculty of Information Technology and Bionics, Pázmány Péter Catholic UniversityBudapest, Hungary

**Keywords:** GnRH neuron, negative estrogen feedback, estrogen receptor beta, GABA, 2-AG, retrograde signaling, CB1

## Abstract

Gonadotropin-releasing hormone (GnRH) neurons are controlled by 17β-estradiol (E2) contributing to the steroid feedback regulation of the reproductive axis. In rodents, E2 exerts a negative feedback effect upon GnRH neurons throughout the estrus-diestrus phase of the ovarian cycle. The present study was undertaken to reveal the role of estrogen receptor subtypes in the mediation of the E2 signal and elucidate the downstream molecular machinery of suppression. The effect of E2 administration at low physiological concentration (10 pM) on GnRH neurons in acute brain slices obtained from metestrous GnRH-green fluorescent protein (GFP) mice was studied under paradigms of blocking or activating estrogen receptor subtypes and interfering with retrograde 2-arachidonoylglycerol (2-AG) signaling. Whole-cell patch clamp recordings revealed that E2 significantly diminished the frequency of spontaneous postsynaptic currents (sPSCs) in GnRH neurons (49.62 ± 7.6%) which effect was abolished by application of the estrogen receptor (ER) α/β blocker Faslodex (1 μM). Pretreatment of the brain slices with cannabinoid receptor type 1 (CB1) inverse agonist AM251 (1 μM) and intracellularly applied endocannabinoid synthesis blocker THL (10 μM) significantly attenuated the effect of E2 on the sPSCs. E2 remained effective in the presence of tetrodotoxin (TTX) indicating a direct action of E2 on GnRH cells. The ERβ specific agonist DPN (10 pM) also significantly decreased the frequency of miniature postsynaptic currents (mPSCs) in GnRH neurons. In addition, the suppressive effect of E2 was completely blocked by the selective ERβ antagonist PHTPP (1 μM) indicating that ERβ is required for the observed rapid effect of the E2. In contrast, the ERα agonist PPT (10 pM) or the membrane-associated G protein-coupled estrogen receptor (GPR30) agonist G1 (10 pM) had no significant effect on the frequency of mPSCs in these neurons. AM251 and tetrahydrolipstatin (THL) significantly abolished the effect of E2 whereas AM251 eliminated the action of DPN on the mPSCs. These data suggest the involvement of the retrograde endocannabinoid mechanism in the rapid direct effect of E2. These results collectively indicate that estrogen receptor beta and 2-AG/CB1 signaling mechanisms are coupled and play an important role in the mediation of the negative estradiol feedback on GnRH neurons in acute slice preparation obtained from intact, metestrous mice.

## Introduction

Hypothalamic gonadotropin-releasing hormone (GnRH) neurons are key regulators of reproduction (Cattanach et al., 1977). They release GnRH into the portal circulation whereby stimulate gonadotropin synthesis and gonadal hormone production (Herbison, 1998). In female rodents, 17β-estradiol (E2) is a main regulator of GnRH neuronal functions (Herbison, 1998). E2 is known to modulate GnRH output via positive and negative feedback mechanisms (Ojeda et al., 1975; Nakai et al., 1978; Attardi et al., 1980; Radovick et al., 2012) and these effects are mediated by estrogen receptors (ERs). GnRH neurons express exclusively estrogen receptor beta (Hrabovszky et al., 2001, 2007; Kalló et al., 2001). The other, alpha subtype of classical estrogen receptor also plays an important role in steroid feedback effects to GnRH neurons, however, this receptor subtype was found exclusively in various synaptic afferent systems, but not in GnRH neurons (Couse and Korach, 1999; Simonian et al., 1999; Wersinger et al., 1999; Couse et al., 2003; Cheong et al., 2014; Yeo and Herbison, 2014; Dubois et al., 2015).

Both ERα and β as transcription factors are located mainly in the nuclei of various hypothalamic neurons where activation of them by E2 triggers classical genomic actions regulating gene transcription (Marino et al., 2006; Radovick et al., 2012). Nevertheless, E2 also exerts rapid, non-genomic actions activating intracellular signaling pathways through extranuclear ERα, ERβ, and G protein-coupled estrogen receptor (GPR30; Abe et al., 2008; Kelly and Rønnekleiv, 2012; Radovick et al., 2012; Kwakowsky et al., 2014). This rapid action of estradiol on GnRH neurons also effectively modulates their functions. E2 administration at low physiological concentration has been published to suppress the firing rate of GnRH neurons with involvement of fast neurotransmission (Chu et al., 2009). The main regulatory neurotransmitter to GnRH neurons is γ-aminobutyric acid (GABA) which is also an important player in the steroid feedback control of GnRH neurons (Sullivan and Moenter, 2003; Moenter and DeFazio, 2005; Christian and Moenter, 2007; Chen and Moenter, 2009; Penatti et al., 2010; Watanabe et al., 2014). GABAergic input received by GnRH neurons is excitatory due to the elevated intracellular chloride level in these cells (DeFazio et al., 2002; Yin et al., 2008; Herbison and Moenter, 2011; Watanabe et al., 2014; Taylor-Burds et al., 2015). Thus, it was also plausible to suppose the involvement of GABA in the suppression of firing of GnRH neurons upon E2 administration (Chu et al., 2009). Nevertheless, activity of GABAergic afferents reaching GnRH neurons can be modulated by the retrograde endocannabinoid signaling mechanisms regulating GABA release from cannabinoid receptor type 1 (CB1) containing axon terminals (Farkas et al., 2010).

In the present study, we hypothesized that the suppressing effect of E2 applied at low physiological concentration on GnRH neurons requires the activation of ERβ and retrograde endocannabinoid signaling mechanisms resulting in the repression of GABAergic neurotransmission onto these neurons. To test this hypothesis, a series of electrophysiological studies has been carried out in GnRH-green fluorescent protein (GFP) neurons of acute slices obtained from intact, metestrous female mice.

## Materials and Methods

### Ethics Statement

All studies were carried out with permissions from the Animal Welfare Committee of the Institute of Experimental Medicine (IEM) Hungarian Academy of Sciences (Permission Number: A5769-01) and in accordance with legal requirements of the European Community (Decree86/609/EEC). All animal experimentation described was conducted in accord with accepted standards of humane animal care and all efforts were made to minimize suffering.

### Experimental Animals

Adult, gonadally intact GnRH-GFP transgenic female mice with C57BL/6J genetic background were used for electrophysiological experiments. In this animal model, a GnRH promoter segment drives selective GFP expression in the majority of GnRH neurons (Suter et al., 2000). Phase of the estrous cycle was checked by both evaluating vaginal smears (Nelson et al., 1982; Caligioni, 2009; Byers et al., 2012) and visual observation of the vaginal opening using the method elaborated recently (Caligioni, 2009; Byers et al., 2012). Mice were used from local colonies bred at the Medical Gene Technology Unit of the IEM. They were maintained in 12 h light/dark cycle (lights on at 06:00 h) and temperature controlled environment (22 ± 2°C), with standard rodent chow and tap water available *ad libitum*. All mice were housed in the same room under same environmental conditions.

### Brain Slice Preparation and Recording

Metestrous mice were deeply anesthetized by Isoflurane inhalation. All mice were killed between 9 a.m. and 10 a.m. and all recordings performed between 1 p.m. and 3 p.m. time. After decapitation, brain was removed rapidly and immersed in ice-cold cutting solution, which had been extensively bubbled with a mixture of 95% O_2_ and 5% CO_2_. The solution contained the following (in mM): saccharose 205, KCl 2.5, NaHCO_3_ 26, MgCl_2_ 5, NaH_2_PO_4_ 1.25, CaCl_2_ 1, and glucose 10. Forebrain blocks were dissected and 250 μm-thick coronal slices were prepared from the medial septum/preoptic area (POA) with a VT-1000S Vibratome (Leica GmBH, Wetzlar, Germany) and placed in the ice-cold oxygenated cutting solution. The slices containing POA were transferred into artificial cerebrospinal fluid (aCSF, in mM: NaCl 130, KCl 3.5, NaH_2_PO_4_ 1.25, MgSO_4_ 1.2, CaCl_2_ 2.5, NaHCO_3_ 26, glucose 10) saturated with O_2_/CO_2_ and kept in it for 1 h to equilibrate. Equilibration started at 33°C and was allowed to cool to room temperature. Electrophysiological recordings were carried out at 33°C, during which the brain slices were oxygenated by bubbling the aCSF with O_2_/CO_2_. Axopatch 200B patch clamp amplifier, Digidata-1322A data acquisition system, and pCLAMP 10.4 software (Molecular Devices Co., Sunnyvale, CA, USA) were used for recording. Cells were visualized with a BX51WI IR-DIC microscope (Olympus Co., Tokyo, Japan) located on an anti-vibration table (Supertech Kft, Pécs, Hungary). The patch electrodes (OD = 1.5 mm, thin wall; Hilgenberg GmbH, Malsfeld, Germany) were pulled with a Flaming-Brown P-97 puller (Sutter Instrument Co., Novato, CA, USA) and polished with an MF-830 microforge (Narishige, Tokyo, Japan). GnRH-GFP neurons were identified by brief illumination at 470 nm using an epifluorescent filter set, based on their green fluorescence, typical fusiform shape, and topographic location in the POA (Suter et al., 2000). After control recording (5 min), the slices were treated with various drugs and the recording continued for a subsequent 10 min.

### Loose-Patch Experiments

Recording of action current firing of GnRH neurons was carried out at 33°C. Pipette potential was 0 mV, pipette resistance 1–2 MΩ, and resistance of loose-patch seal 7–40 MΩ. The pipette solution contained (in mM): NaCl 150, KCl 3.5, CaCl_2_ 2.5, MgCl_2_ 1.3, HEPES 10, and glucose 10 (pH 7.3).

After recording basal action currents, the E2 (10 pM; Sigma) was added in a single bolus to the brain slice in the recording chamber, and the recording continued for a subsequent 10 min.

### Whole-Cell Patch Clamp Experiments

The cells were voltage clamped at −70 mV holding potential. Pipette offset potential, series resistance (R_s_) and capacitance were compensated before recording. Only cells with low holding current (<50 pA) and stable baseline were used. Input resistance (R_in_), R_s_, and membrane capacity (C_m_) were also measured before each recording by using 5 mV hyperpolarizing pulses. To ensure consistent recording qualities, only cells with R_s_ < 20 MΩ, R_in_ > 500 MΩ, and C_m_ > 10 pF were accepted. The intracellular pipette solution contained (in mM): HEPES 10, KCl 140, EGTA 5, CaCl_2_ 0.1, Mg-ATP 4, and Na-GTP 0.4 (pH 7.3). The resistance of the patch electrodes was 2–3 MΩ. The spontaneous postsynaptic currents (sPSCs) measurements were carried out with an initial control recording (5 min), then low physiological dose of E2 (10pM; Nelson et al., 1992; Christian et al., 2005; Freeman, 2006; Chu et al., 2009) was added to the aCSF in the recording chamber and the recording continued for a subsequent 10 min. When the CB1 inverse agonist AM251 (N-(Piperidin-1-yl)-5-(4-iodophenyl)-1-(2, 4-dichlorophenyl)-4-methyl-1H-pyrazole-3-carboxamide; 1 μM; Tocris; Farkas et al., 2010, 2013; Lee et al., 2015), the estrogen receptor antagonist Faslodex/ICI 182,780 (7α, 17β-[9-[(4, 4, 5, 5, 5-Pentafluoropentyl)sulfinyl]nonyl]estra-1, 3, 5(10)-triene-3, 17-diol; 1 μM; Tocris; Chu et al., 2009; Farkas et al., 2010) or the ERβ antagonist PHTPP (4-[2-Phenyl-5,7-bis(trifluoromethyl)pyrazolo[1,5-a]pyrimidin-3-yl]phenol; 1 μM; Tocris; Kajta et al., 2013; Saleh et al., 2013) were used, they were added to the aCSF 10 min before starting the recording. The diacylglycerol (DAG) lipase inhibitor tetrahydrolipstatin (THL, N-Formyl-L-leucine (1S)-1-[[(2S, 3S)-3-hexyl-4-oxo-2-oxetanyl]methyl]dodecyl ester; 10 μM; Tocris; Farkas et al., 2010, 2013) was added to the intracellular solution in the pipette to block 2-AG synthesis. To minimize THL spill, the GnRH cells were approached rapidly (<1 min), and the flow rate of aCSF was increased from 5–6 to 8–9 ml/min. Just before release of the positive pressure in the pipette, the flow rate was restored to 5–6 ml/min to avoid any mechanical movement of the slice. The pipette solution containing THL was allowed to equilibrate with the intracellular milieu of the cell for 15 min before starting recording. For miniature postsynaptic current (mPSCs) recordings 10 min before start the spike-mediated transmitter release was blocked by adding the voltage sensitive Na-channel inhibitor tetrodotoxin (TTX; 646 nM; Tocris) to the aCSF. After 5 min control recording E2 (10 pM), the selective ERα agonist PPT (4,4′,4″-(4-Propyl-[1H]-pyrazole-1,3,5-triyl) trisphenol; 10 pM; Tocris), the selective ERβ agonist DPN (2,3-bis(4-Hydroxyphenyl)-propionitrile; 10 pM; Tocris) or the selective GPR30 receptor agonist G1 ((±)-1-[(3aR*,4S*,9bS*)-4-(6-Bromo-1,3-benzodioxol-5-yl)-3a,4,5,9b-tetrahydro 3H cyclopenta [c] quinolin-8-yl]- ethanone; 10 pM; Tocris) was added to the aCSF respectively, and the recording continued for a subsequent 10 min. E2, PPT, DPN and G1 were pipetted onto the slice in a single bolus. In order to keep comparability, all ER agonists were used at the same 10 pM concentration.

### Statistical Analysis

Each experimental group contained 8–18 recorded cells from six to nine animals. Responding cells were defined according to definition of Chu et al. (2009) with modification: cells were considered as responding ones if any negative change was detected in their frequency. Recordings were stored and analyzed off-line. Mean firing rate and mPSC frequency were calculated as number of spikes divided by the length of the respective period (5 min “baseline value” and 10 min “agonist period”, respectively). Percentage changes resulting from drugs were calculated by dividing the value to be analyzed before (5 min) and after (the subsequent 10 min) respective agonist administration. Each neuron served as its own control when drug effects were evaluated. Event detection was performed using the Clampfit module of the PClamp 10.4 software (Molecular Devices Co.). Group data were expressed as mean ± SEM and percentage change in the frequency of the PSCs due to the application of various drugs was calculated. Statistical analyses were carried out using Prism 3.0 (GraphPad Software, Inc., GraphPad). Statistical significance was analyzed using Kruskal-Wallis test followed by Dunns post-test for comparison of groups whereas cumulative probabilities were analyzed with Kolmogorov-Smirnov test and considered as significant at *p* < 0.05.

## Results

### E2 Significantly Decreases the Firing Rate and Frequency of sPSCs in GnRH Neurons of Metestrous Female Mice

**Figure 1:**
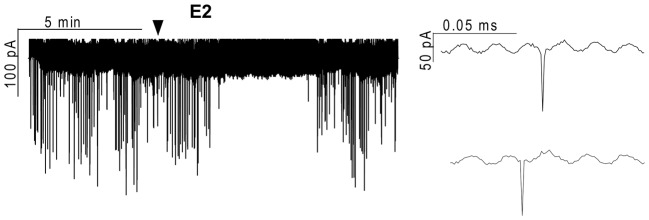
**Effect of 17β-estradiol (E2) on the action currents of gonadotropin-releasing hormone (GnRH) neurons in brain slice of metestrous female mouse.** Application of 10 pM E2 resulted in a significant decrease in the frequency of the action currents on GnRH neurons. Individual events during the control phase (upper inset) and the E2-treated phase (lower inset) present no change. Arrowhead shows the onset of E2 administration.

**Figure 2:**
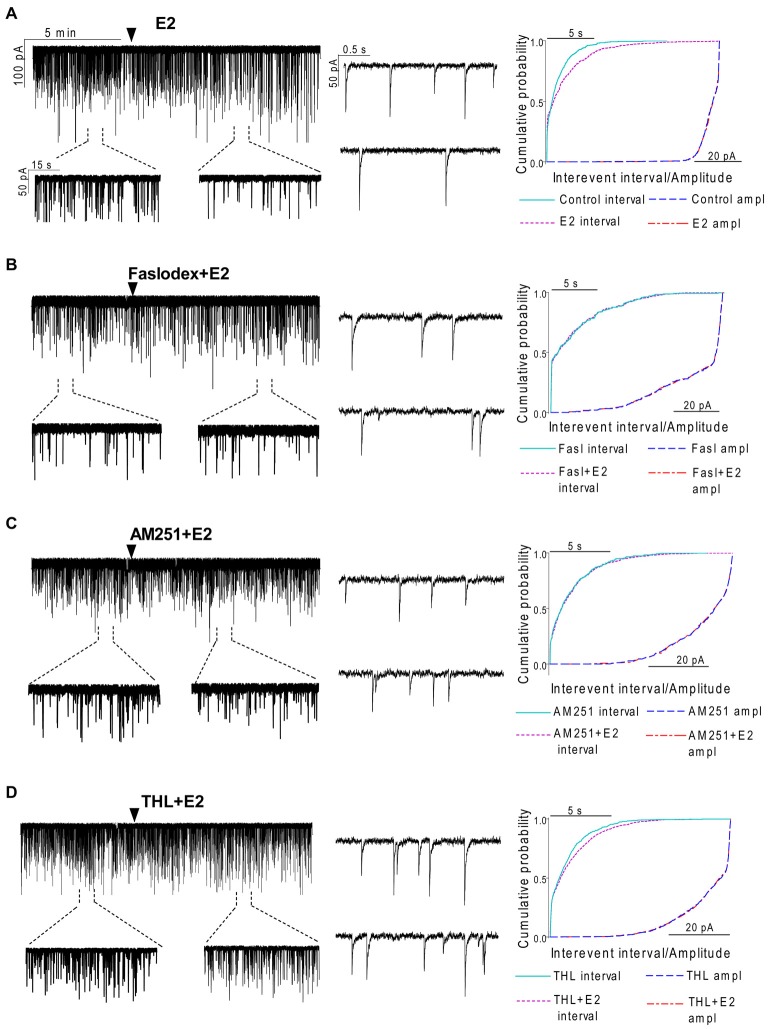
**Effect of E2 on the spontaneous postsynaptic currents (sPSCs) of GnRH neurons in brain slice of metestrous female mice. (A)** E2 in low concentration (10 pM) decreased the frequency of the sPSCs with no change in the average amplitude of them. One-minute-long periods of the recording before and after application of E2 are depicted under the recording. Cumulative probability plot presents reduction in the interevent intervals, but not in amplitude. **(B)** Pretreatment of the brain slice with the non-selective ER antagonist Faslodex (1 μM, 10 min) inhibited the effect of E2 on the sPSCs. **(C)** Effect of E2 on the sPSCs was abolished by the pretreatment with cannabinoid receptor type 1 (CB1) inverse agonist AM251 (1 μM, 10 min). **(D)** Similar inhibition was observed in case of intracellularly applied diacylglycerol (DAG) lipase inhibitor THL (10 μM, 20 min). Individual events of sPSCs show no change in waveform properties in the treated phase (lower insets) as compared to the control phase (upper insets) in all sPSC measurements. Cumulative probability plots in **(B–D)** graphs present no change in interevent intervals and amplitudes. Arrowhead shows the onset of drug administration.

In order to examine the action of E2 on GnRH neurons of metestrous female mice, first, loose-patch studies were carried out. At low physiological concentration (10 pM) E2 decreased the firing activity of GnRH neurons (Figure 1) in accordance with the original finding of Chu et al. (2009). Then sPSCs were recorded to demonstrate the action of E2 in GnRH neurons using whole-cell patch clamp method. The mean stochastic change in the frequency of the non-treated “responding” GnRH neurons was 83.72 ± 3.8% which was used later as control value for the statistical analysis. Administration of E2 (10 pM) resulted in a significant decrease in the sPSCs in 9 of 18 of examined GnRH neurons (49.62 ± 7.6% of the baseline value 1.26 ± 0.4 Hz; *p* < 0.05; Figures 2A, 6), whereas the amplitude and decay of the sPSCs exhibited no change (Figure 6, Table 1) suggesting role of a presynaptic process. E2 decreased the frequency of sPSCs within 1–2 min indicating that this phenomenon was due to the rapid, non-genomic effect of E2. This action was blocked by the non-selective ER antagonist Faslodex (1 μM; Figures 2B, 6). In the presence of Faslodex, after E2 application the mean frequency of sPSCs (77.80 ± 5.8% of the baseline values, 2.48 ± 0.8 Hz; *n* = 7) was significantly higher (*p* < 0.05) compared to the percentage change in the frequency of sPSCs in the case of E2 alone (Figure 6). This result indicated that E2 utilized estrogen receptor(s) in this rapid effect.

**Table 1:** **Neither the amplitude nor the decay phase of the spontaneous postsynaptic currents (sPSCs) showed significant change after the various treatments**.

	Amplitude (control; pA)	Amplitude change (% of the control)	Decay tau (control; ms)	Decay tau change (% of the control)
E2	−37.0 ± 5.0	104.7 ± 3.6	14.9 ± 1.9	129.9 ± 12.4
Faslodex+E2	−33.7 ± 3.9	99.3 ± 4.3	25.4 ± 5.6	148.5 ± 29.5
AM251+E2	−37.4 ± 6.8	112.6 ± 14.1	23.6 ± 4.1	153.9 ± 33.1
THL+E2	−36.3 ± 4.1	101.1 ± 3.6	16.2 ± 1.4	130.3 ± 17.2

*The table shows the mean amplitude and decay tau parameters before drug administration and the percentage change in mean amplitudes and decay tau resulting from the drug application*.

### Endocannabinoid 2-AG Signaling is Involved in E2-Triggered Decrease of sPSC Frequency

One of our earlier studies showed that endocannabinoid release from GnRH neuron was able to influence synaptic transmission to the GnRH neuron itself (Farkas et al., 2010). Thus, the CB1 inverse agonist AM251 (1 μM) was used to test the putative role of the retrograde endocannabinoid signaling mechanism in the mediation of the effect of E2 on GnRH neurons. AM251 pretreatment attenuated the effect of E2 on the frequency of sPSCs (86.00 ± 4.1% of the baseline value 1.99 ± 0.5 Hz; *n* = 6) on GnRH neurons (Figures 2C, 6), with no change in amplitude (Figure 6, Table 1), supporting the hypothesis that endocannabinoids were involved in E2-evoked decrease of sPSC frequency. There are two main types of endocannabinoids in the CNS, anandamide and 2-arachidonoylglycerol (2-AG). Diacylglycerol lipase (DAGL) is the 2-AG synthesizing enzyme, thus the selective inhibitor of DAGL, THL, was used to clarify which type of endocannabinoid was involved in the acute effect of E2 on GnRH neurons. The intracellularly applied THL (10 μM) diminished the effect of E2 on the frequency of sPSCs (67.61 ± 5.8% of baseline value 2.18 ± 0.4 Hz; *n* = 9; Figures 2D, 6), with no change in amplitude (Figure 6, Table 1) indicating that 2-AG synthesized by GnRH neurons was involved in the action of E2.

### Effect of E2 is Direct on GnRH Neurons of Metestrous Female Mice

**Figure 3:**
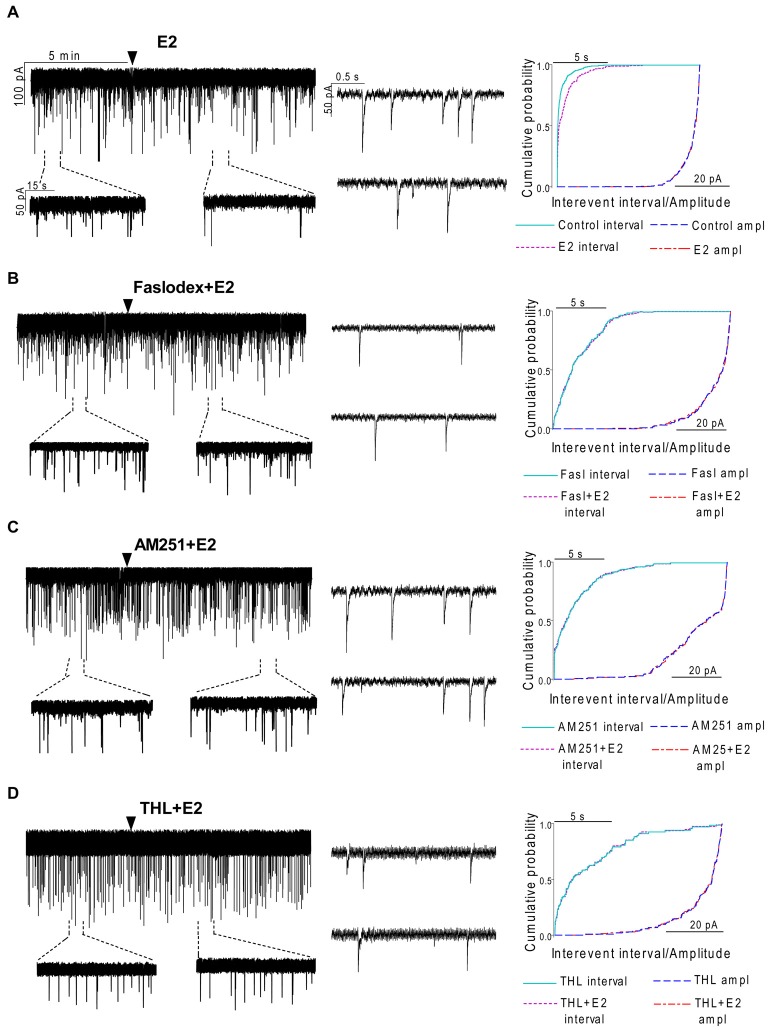
**Effect of E2 on the miniature postsynaptic currents (mPSCs) of GnRH neurons in the presence of non-selective estrogen receptor (ER) antagonist and endocannabinoid receptor/synthesis blockers. (A)** E2 decreased the frequency of the mPSCs with no change in the average amplitude of them. One-minute-long periods of the recording before and after application of E2 are illustrated under the recording. Cumulative probability plot presents reduction in the interevent intervals, but not in amplitude in the case of the effect of E2 alone on the mPSCs. **(B)** Pretreatment of the brain slice with the non-selective ER antagonist Faslodex (1 μM, 10 min) inhibited the action of E2 on the mPSCs. **(C)** Effect of E2 on the mPSCs was abolished by pretreatment with cannabinoid receptor type 1 (CB1) inverse agonist AM251 (1 μM, 10 min). **(D)** Similar inhibition was observed in case of intracellularly applied DAG lipase inhibitor THL (10 μM, 20 min). Arrowhead shows the onset of drug administration. Individual events of mPSC in the control phase (upper insets) and the treated phase (lower insets) show no change in waveform properties in any measurements **(A—D)**. Cumulative probability plots of treatments in **(B–D)** graphs show no change in interevent intervals or amplitudes.

To examine the putative direct effect of E2 on GnRH neurons, miniature postsynaptic currents (mPSCs) were recorded in the presence of TTX (646 nM) to inhibit propagation of action potentials during whole-cell patch clamp recording. TTX was used in all subsequent measurements. The excitatory GABA is a major mediator of fast synaptic transmission via GABA_A_-R on GnRH neurons and the recorded mPSCs observed under the circumstances used in our experiments were exclusively GABAergic (Sullivan and Moenter, 2003; Moenter and DeFazio, 2005; Yin et al., 2008; Farkas et al., 2010; Herbison and Moenter, 2011). Administration of E2 (10 pM) caused a significant decrease in the mean frequency of the GABAergic mPSCs in GnRH neurons (8 of 12 examined neurons). Frequency of the mPSCs declined to 50.75 ± 9.6% (compared to the baseline value 2.29 ± 0.4 Hz; *p* < 0.05; Figures 3A, 7), while amplitude and decay of the mPSCs showed no significant alteration (Figure 7, Table 2).

**Table 2:** **Neither the amplitude nor the decay phase of the miniature postsynaptic currents (mPSCs) showed significant change after the various treatments**.

	Amplitude (control; pA)	Amplitude change (% of the control)	Decay tau (control; ms)	Decay tau change (% of the control)
E2	−31.6 ± 3.0	102.4 ± 5.3	9.7 ± 1.7	103.2 ± 18.1
Faslodex+E2	−27.8 ± 2.4	103.0 ± 2.2	7.1 ± 0.9	95.9 ± 20.1
AM251+E2	−24.7 ± 2.2	101.3 ± 3.5	8.6 ± 2.6	103.3 ± 28.5
THL+E2	−30.0 ± 4.3	93.8 ± 3.4	5.7 ± 1.2	104.7 ± 29.2
DPN	−36.6 ± 6.7	102.9 ± 5.1	14.1 ± 2.2	114.5 ± 11.3
PHTPP+E2	−26.6 ± 3.0	100.1 ± 4.0	9.5 ± 1.2	102.4 ± 22.4
AM251+DPN	−32.7 ± 10.6	97.1 ± 6.0	6.6 ± 1.3	128.4 ± 26.6
PPT	−28.7 ± 3.1	98.7 ± 6.7	12.9 ± 1.6	108.2 ± 15.8
G1	−33.0 ± 2.6	98.8 ± 2.2	6.0 ± 0.7	116.0 ± 19.3

*The table shows the mean amplitude and decay tau before drug administration and the percentage change in these parameters resulting from the drug administrations*.

### ERβ is Required for the Rapid Effect of E2 on GnRH Neurons of Metestrous Female Mice

**Figure 4:**
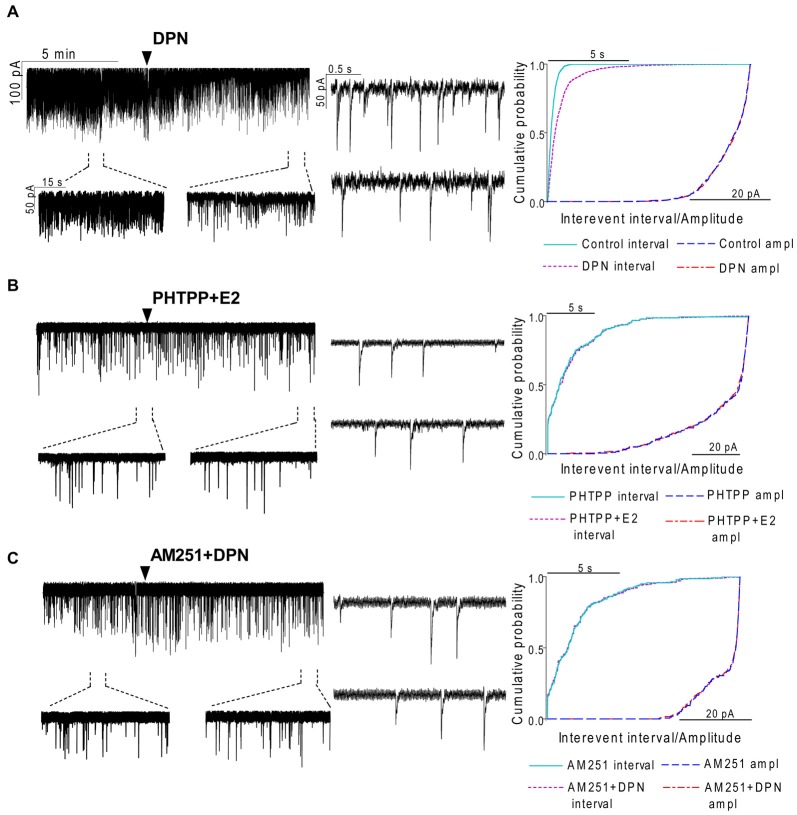
**The effect of ERβ activation on the mPSCs in GnRH neurons of the metestrous female mice: outcome of ERβ antagonist, agonist and CB1 blocker. (A)** The subtype-selective ERβ agonist DPN (10 pM, 10 min) significantly decreased the frequency of mPSCs. One-minute long periods of the recording before and after application of the agonist are illustrated under the recording. Cumulative probability plot presents reduction of the interevent intervals, but not in amplitude. **(B)** Pretreatment of the brain slice with the selective ERβ receptor antagonist PHTPP (1 μM, 10 min) inhibited the effect of E2 on mPSCs.** (C)** ERβ agonist DPN had no significant effect on the frequency of mPSCs in the presence of AM251 (1 μM, 10 min). Arrowhead shows the onset of drug administration. Individual events of mPSC show no change in waveform properties of the treated phase (lower insets) as compared to the control phase (upper insets) in any of the mPSC measurements. Cumulative probability plots show no change in interevent intervals and amplitudes in **(B,C)** graphs.

**Figure 5:**
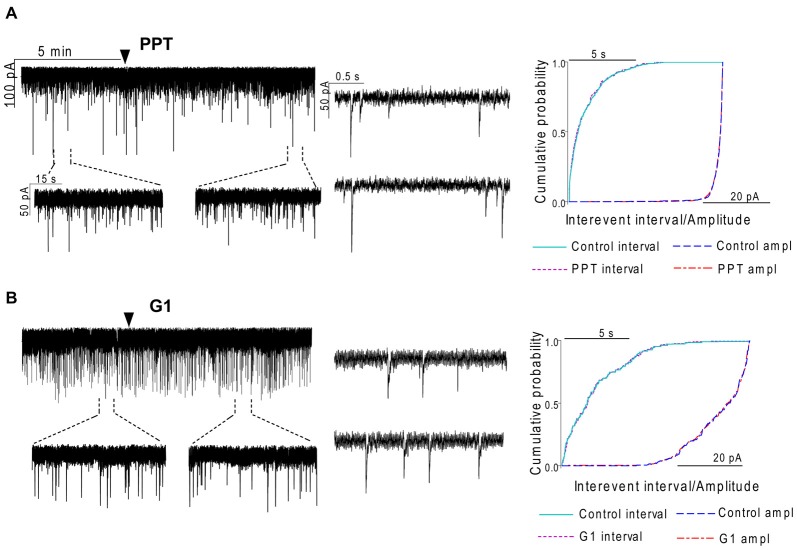
**The selective ERα agonist and G protein-coupled estrogen receptor (GPR30) agonist exert no effects on the mPSCs in GnRH neurons of the metestrous female mice. (A)** The selective ERα agonist PPT (10 pM, 10 min) was unable to modify the frequency of mPSCs in the recorded GnRH neurons. **(B)** Similarly, the GPR30 receptor agonist G1 (10 pM; 10 min) did not modify the frequency of the mPSCs. Arrowhead shows the onset of drug administration. Individual events of mPSC show no change in waveform properties of the treated phase (lower insets) compared to the control phase (upper insets) in each mPSC measurements. Cumulative probability plots of the treatments show no change in interevent intervals or amplitudes.

In order to demonstrate involvement of the ERs in the direct action of E2 on GnRH neurons, the non-selective ER antagonist Faslodex (1 μM) was used. The effect of E2 was blocked by Faslodex (Figure 3B). In the presence of the antagonist, after E2 administration the mean frequency of mPSCs (84.19 ± 4.0% of baseline value 0.53 ± 0.1 Hz; *n* = 6) was significantly higher (*p* < 0.05) compared to the value measured with E2 alone (Figure 7, Table 2). Since the involvement of ER subtypes in the negative feedback effect of E2 has not been clarified yet, we used subtype-selective ER agonists to identify the putative role of ERα and/or ERβ in the mediation of E2 effect on GnRH neurons. The ERβ agonist DPN (10 pM) significantly decreased the mean frequency of the mPSCs in GnRH neurons (60.65 ± 5.1% compared to the baseline value 2.16 ± 0.6 Hz; *n* = 8; *p* < 0.05; Figures 4A, 8). In line with this observation, the effect of E2 was significantly blocked (73.02 ± 6.1% of baseline value 0.68 ± 0.1 Hz; *n* = 7; *p* < 0.05) by the specific ERβ antagonist PHTPP (1 μM) administration (Figures 4B, 8). These results indicate that ERβ is required for the observed rapid effect of E2 in GnRH neurons. In contrast, the ERα agonist PPT (10 pM) had no significant effect on the frequency of mPSCs in GnRH neurons (78.70 ± 6.4% of baseline value 2.32 ± 1.2 Hz; *n* = 7; *p* > 0.05; Figures 5A, 8). We also addressed the putative role of the membrane associated GPR30 in this process. Application of the GPR30 selective agonist G1 (10 pM) had no significant effect on the frequency of the mPSCs (86.05 ± 3.5% as compared to the baselined value 0.38 ± 0.1 Hz; *n* = 5; *p* > 0.05; Figures 5B, 8). The amplitude and decay tau of the mPSCs did not change upon any of these treatments (Figure 8, Table 2). These data show that ERα and GPR30 have no role in mediating the observed rapid effect of the E2 on GnRH neurons.

**Figure 6:**
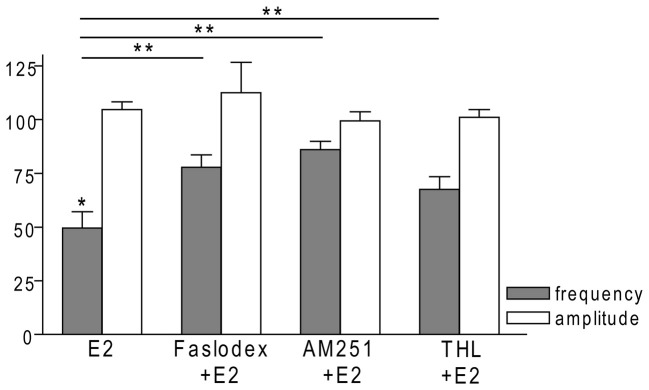
**Bar graph summarizing the percentage changes in the frequency and the amplitude of the sPSCs resulting from E2 treatment in the presence of Faslodex, AM251 and THL.** E2 significantly decreased the frequency of sPSCs. Inhibition of its effect could be achieved with antagonizing the ERs, CB1 receptors or blocking the intracellular 2-AG endocannabinoid synthesis. The amplitude of the mPSCs did not change in any of the treatments. *p < 0.05 as compared to the control; **p < 0.05 as compared to the change evoked by E2 treatment.

**Figure 7:**
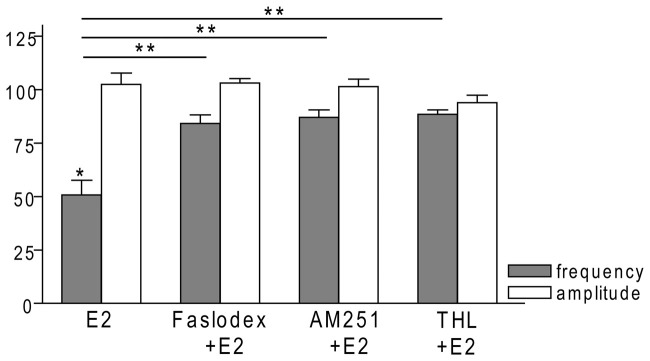
**Bar graph summarizing the percentage changes in the frequency and the amplitude of the mPSCs resulting from E2 treatment in the presence of Faslodex, AM251 and THL.** E2 significantly decreased the frequency of mPSCs. Inhibition of this effect could be achieved with antagonizing the ERs by Faslodex. Effect of E2 was eliminated by the pretreatment with CB1 inverse agonist AM251 or the intracellularly applied 2-AG endocannabinoid synthesis blocker THL. The amplitude of the mPSCs did not change in any of the treatments. *p < 0.05 as compared to the control; **p < 0.05 as compared to the change evoked by E2 treatment.

**Figure 8:**
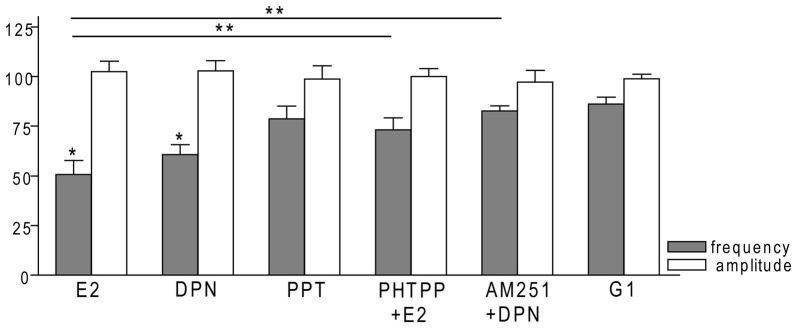
**Bar graph summarizing the percentage changes in the frequency and the amplitude of the mPSCs resulting from selective ER agonists and various antagonists.** The E2 and the selective ERβ agonist DPN significantly decreased the frequency of mPSCs. Effect of DPN was eliminated by the pretreatment with CB1 inverse agonist AM251. Effect of E2 could be inhibited by antagonizing selectively the ERβ by PHTPP. The selective ERα agonist PPT and the GPR30 receptor agonist G1 had no significant effect on the frequency of mPSCs. The amplitude of the mPSCs presented no change in any of the treatments. *p < 0.05 compare to the control; **p < 0.05 as compared to the change caused by E2 treatment.

### Effect of E2 on the mPSCs of GnRH Neurons is Mediated via Activation of 2-AG Retrograde Endocannabinoid Signaling Mechanism

In order to demonstrate that E2 (10 pM) decreases GnRH neuron mPSC activity via a retrograde endocannabinoid mechanism AM251 was added to the aCSF. AM251 eliminated the action of E2 on the GABAergic mPSCs in a significant manner (86.99 ± 3.5% of baseline value 0.87 ± 0.2 Hz; *n* = 5; *p* < 0.05; Figures 3C, 7). Similar effect was observed when DPN (10 pM) was used in the presence of AM251 (82.56 ± 2.6% of baseline value 1.08 ± 0.3 Hz; *n* = 5; Figures 4C, 8). The intracellularly applied THL also eliminated the action of E2 on mPSCs (88.39 ± 2.0% of baseline value 0.77 ± 0.2 Hz; *n* = 5; *p* < 0.05; Figures 3D, 7). The amplitude and decay tau of the mPSCs did not change after these treatments (Figures 7, 8, Table 2). These results support the idea that the retrograde endocannabinoid signaling mechanism was involved in the suppression of GnRH activity when low physiological concentration of estradiol was used.

## Discussion

The present study provides electrophysiological evidence for the involvement of ERβ and 2-AG signaling in the mediation of the effect of E2 suppressing fast neurotransmission onto GnRH neurons in metestrous female mice. Accordingly, (1) E2 significantly decreases the firing rate and frequency of sPSCs and mPSCs in GnRH neurons; (2) This direct and rapid effect of E2 requires the cooperation of ERβ in GnRH neurons; and (3) The retrograde endocannabinoid 2-AG signaling is involved in the E2-triggered decrease of sPSC and mPSC frequency of GnRH neurons.

### E2 Significantly Decreases the Firing Rate and Frequency of sPSCs and mPSCs in GnRH Neurons in Metestrous Female Mice

Our results showed that both firing rate and the GABAergic neurotransmission to GnRH neurons were inhibited by low physiological dose of E2. These data support earlier findings showing that E2 at 10 pM concentration is able to diminish the firing of GnRH neurons (Chu et al., 2009). In the hypothalamus, estradiol has also been reported to suppress the neurokinin-B agonist (senktide)-induced firing rate in kisspeptin/neurokinin-B/dynorphin (KNDY) neurons of the arcuate nucleus (Simonian et al., 1999). Regarding extrahypothalamic actions, spontaneous firing activity of neurons in the lateral habenula was also inhibited by estrogen (Kokay et al., 2011). In addition, our data also demonstrated that frequency of the GABAergic postsynaptic currents was decreased upon E2 treatment in GnRH neurons of metestrous female mice. Not surprisingly, neurons of other brain regions, such as hippocampus, exhibit decreased PSC frequency upon estradiol treatment (Huang and Woolley, 2012; Tabatadze et al., 2015). Kisspeptin neurons of the arcuate nucleus were also shown to respond to E2 administration with a reduced mPSC frequency (DeFazio et al., 2014). Our results, therefore, is in a good agreement with the earlier results, revealing positive correlation between firing rate and frequency of postsynaptic currents in GnRH neurons (Chu and Moenter, 2005; Christian and Moenter, 2007; Farkas et al., 2013).

### The Execution of Direct, Rapid Effect of E2 Requires ERβ in GnRH Neurons

Till the late 1990’s, the general consensus has been that E2 modulates GnRH neurons via estrogen-sensing interneurons located in hypothalamic and different extrahypothalamic loci, because earlier studies using autoradiography combined with immunocytochemistry showed that GnRH neurons did not express ER (Shivers et al., 1983), whereas certain neuron sets innervating GnRH neurons contained nuclear ERα (Herbison, 1998; Simonian et al., 1999; Smith et al., 2005; Franceschini et al., 2006). The discovery of ERβ (Kuiper et al., 1996) lead to the finding that ERβ was expressed in GnRH neurons in rodents (Hrabovszky et al., 2000, 2001; Herbison and Pape, 2001; Kalló et al., 2001) and humans (Hrabovszky et al., 2007). Moreover, a broad range of experiments demonstrated that E2 acted directly on GnRH neurons (Abrahám et al., 2003; Petersen et al., 2003; Temple et al., 2004; Abe and Terasawa, 2005; Abe et al., 2008; Chu et al., 2009). In this work, our laboratory extended these studies by examining the direct effect of E2 in GnRH neurons of metestrous female mice. Administration of low physiological concentration (10 pM) of E2 resulted in a significant decrease in the sPSC frequency in GnRH neurons within 1–2 min. This action was inhibited by administration of the non-selective estrogen receptor antagonist, Faslodex, indicating the involvement of estrogen receptor(s) in this rapid effect. Furthermore, application of E2 resulted in a significant decrease in the mean frequency of the mPSCs in GnRH neurons indicating that the observed effect of E2 was direct on GnRH neurons. When the 2-AG endocannabinoid synthesis blocker THL was administered intracellularly, it eliminated the effect of E2 on mPSCs confirming further that the effect of E2 on GnRH neurons was direct. In addition, this action of E2 was rapid, in the range of a few minutes, suggesting activation of intracellular signaling pathways via membrane-associated receptors, such as ERα, ERβ, and the GPR30 (Abe et al., 2008; Kelly and Rønnekleiv, 2012; Radovick et al., 2012; Kwakowsky et al., 2014).

In order to identify the exact subtype of the ER mediating this rapid E2 action in GnRH neurons, we examined the effect of various subtype-selective ER agonists. The ERβ agonist DPN significantly decreased the mean frequency of the mPSCs in GnRH neurons. Moreover, the effect of E2 was significantly blocked by the ERβ specific antagonist PHTPP. In contrast, neither the ERα agonist PPT nor the GPR30 selective agonist G1 had significant effect. These findings indicate that ERβ is required exclusively for the observed rapid effects of E2 in GnRH neurons. In line with this observation, *in vivo* studies in ovariectomized mice showed that GnRH neurons responded to estrogen in a rapid and direct manner through an ERβ-dependent mechanism (Abrahám et al., 2003). Our present experiments provided further evidence about the pivotal role of ERβ in the mediation of the rapid effect of E2 in GnRH neurons in acute slice preparation from metestrous female mice during the negative estrogen feedback period.

### Retrograde 2-AG Signaling is Involved in the E2-Triggered Decrease of sPSC and mPSC Frequency in GnRH Neurons

The GABA neurotransmission has been considered as one of the main regulatory signaling to GnRH neurons. Series of studies proved that GABA acts as an excitatory neurotransmitter on postsynaptic GABA_A_-R channels of adult GnRH neurons of rodents (DeFazio et al., 2002; Moenter and DeFazio, 2005; Yin et al., 2008; Watanabe et al., 2009, 2014; Herbison and Moenter, 2011; Taylor-Burds et al., 2015). Furthermore, several experiments demonstrated that the frequency of the GABAergic mPSCs was in a positive correlation with the firing activity of these neurons (Chu and Moenter, 2005; Christian and Moenter, 2007; Chen and Moenter, 2009; Farkas et al., 2010). A previous study from our laboratory demonstrated that the 2-AG release from GnRH neurons resulted in a simultaneous reduction of the firing rate and GABAergic neurotransmission to GnRH neurons via GABA_A_-R. GnRH neurons present a tonic 2-AG production because both AM251 and THL could influence the GABAergic input of these cells (Farkas et al., 2010). Therefore, the E2-triggered decline in the firing rate and the frequency of the mPSCs may suggest the putative involvement of the retrograde endocannabinoid signaling machinery in the manifestation of the suppressing effect of E2 in the metestrus stage. It is in line with a previous report demonstrating that release of endocannabinoids indeed regulated the function of GnRH neurons (Glanowska and Moenter, 2011). Thus, our data that AM251 and the intracellularly applied THL inhibited the effect of the low physiological dose of E2 in the negative feedback period suggest the involvement of retrograde 2-AG signaling mechanism in the achievement of E2-evoked changes observed in the PSCs of the GnRH neuron. The endocannabinoid system has also been described as a downstream element of E2 pathway in adult female rat hippocampus where a principal role of retrograde endocannabinoid signaling in the E2-dependent suppression of inhibitory GABAergic neurotransmission to CA1 pyramidal neurons was elucidated (Huang and Woolley, 2012).

Our intracellular THL administration proved that the E2-evoked 2-AG synthesis took place in GnRH neurons. In line with this, a previous study showed that immortalized GnRH neurons synthesize 2-AG (Gammon et al., 2005) and patch clamp experiments on GnRH neurons in acute brain slices also supported this finding (Farkas et al., 2010; Glanowska and Moenter, 2011). Endocannabinoids are synthesized and released from postsynaptic GnRH neurons upon E2 activation, thereafter, they act in a retrograde manner on CB1 receptors expressed in presynaptic axon terminals innervating GnRH neurons, similarly to other, endocannabinoid-regulated systems (Sugiura and Waku, 2000; Piomelli, 2003; Kano et al., 2009; Ohno-Shosaku and Kano, 2014).

**Figure 9:**
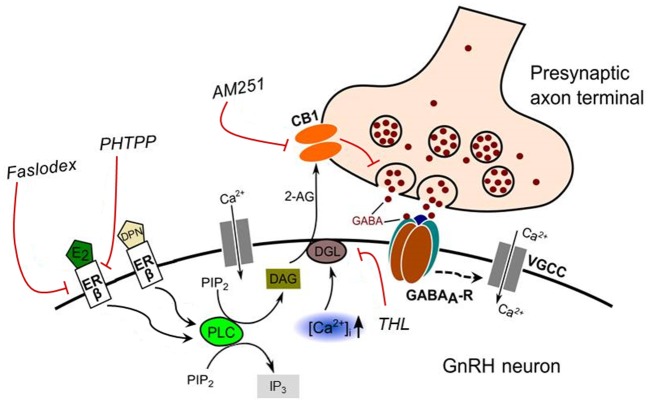
**Schematic illustration of the interaction between E2 and endocannabinoid signaling in GnRH neuron of the metestrous female mice**. Binding of E2 to ERβ activates synthesis and release of 2-AG in the GnRH neuron. The released endocannabinoid 2-AG then binds to CB1 expressed in the presynaptic terminal of GABAergic afferents and causes suppression of GABA release into the synaptic cleft. This effect of E2 was blocked when the non-selective ER antagonist (Faslodex) or the selective ERβ receptor antagonist (PHTPP) was administered. The signaling was also inhibited when the CB1 inverse agonist (AM251) or the DAG lipase inhibitor (THL) was applied. E2, 17β-estradiol; ERβ, estrogen receptor beta; DPN, subtype selective ERβ agonist; DAG, diacylglycerol; DGL, DAG-lipase; CB1, cannabinoid receptor type-1; AM251, CB1 inverse agonist; Faslodex, non-selective estrogen receptor antagonist; PHTPP, subtype selective ERβ antagonist; 2-AG, 2-arachidonoylglycerol; THL, tetrahydrolipstatin (DAG-lipase inhibitor); PIP_2_, phosphatidylinositol 4,5-bisphosphate; IP_3_, inositol 1,4,5-trisphosphate; PLC, phospholipase-C; GABA_A_-R, GABA_A_ receptor; $\left[{\text{Ca}}_{\text{I}}^{2+}\right]$, intracellular free calcium; VGCC, voltage-gated calcium channel. Dashed arrow denote putative action.

The proposed model of E2 action on the GnRH neuron is illustrated in Figure 9.

To sum up, this study suggests that E2 binds to ERβ and triggers the synthesis and release of 2-AG from GnRH neurons. Then, 2-AG binds to CB1 located in the presynaptic terminals of GABAergic afferents, which eventually causes the suppression of GABA release into the synaptic cleft and in turn, the repression of electric activity of GnRH neurons. The elucidation of the putative participation of the ERβ-2-AG signaling mechanism in GnRH neurons of humans raises a further challenge in understanding the pathophysiology of central, hypothalamic regulatory mechanisms of the GnRH neuronal network orchestrating reproduction.

## Author Contributions

FB carried out electrophysiological recordings and data analysis, ZL participated in designing the experiments, IF carried out recordings, data analysis and designing the experiments.

## Conflict of Interest Statement

The authors declare that the research was conducted in the absence of any commercial or financial relationships that could be construed as a potential conflict of interest.
